# A systematic review and meta‐analysis of the linkage between low vitamin D and the risk as well as the prognosis of stroke

**DOI:** 10.1002/brb3.3577

**Published:** 2024-06-14

**Authors:** Jianrong Xiong, Chenliang Zhao, Jinhui Li, Yongxiang Li

**Affiliations:** ^1^ Department of Rehabilitation Medicine Fourth Affiliated Hospital of Zhejiang University Medical College Yiwu Zhejiang China; ^2^ Intensive Care Medicine Heyou Hospital, Foshan Guangzhou China; ^3^ Department of Chinese Medicine & Rehabilitation Medicine The Second Affiliated Hospital of Zhejiang University Medical College Hangzhou Zhejiang China

**Keywords:** meta‐analysis, prognosis, stroke, systematic review, vitamin D

## Abstract

**Objective:**

The research intended to probe the connection between the risk of stroke and serum vitamin D levels.

**Methods:**

Three electronic databases (Cochrane Library, EMBASE, PubMed) were searched according to the subject terms from inception until July 29, 2022, and retrieved researches were screened on the basis of inclusion and exclusion criteria. Two investigators conducted the quality assessment and data extraction. Using Stata 16.0 software, a meta‐analysis was conducted on the extracted data.

**Findings:**

In total, 27 studies with 45,302 participants were included. Among these studies, 20 focused on stroke risk, while 7 examined stroke prognosis. According to the meta‐analysis findings, it was observed that a higher stroke risk is connected to reduced levels of serum vitamin D. This association was reflected in a combined relative risk (RR) of 1 .28 (95% confidence interval (CI): 1.15–1.42) and a worse prognosis after stroke (RR = 2.95, 95% CI: 1.90–4.60). Additional analysis indicated that no apparent relationship between a decrease in vitamin D and the probability of experiencing a hemorrhagic stroke was found. The RR found was 1.93 (95% CI: 0.95–3.95). On the other hand, it was observed that a reduction in serum vitamin D levels was linked to an elevated likelihood of developing an ischemic stroke. The RR identified was 1.72 (95% CI: 1.78–2.03). Moreover, a lower level of vitamin D in the bloodstream was associated with a more unfavorable prognosis for individuals who suffered from a stroke. The RR for this correlation was 2.95 (95% CI: 1.90–4.60). However, further research is required to confirm the above‐mentioned findings.

**Conclusion:**

In conclusion, lower concentration vitamin D was found to be related to an increased risk of stroke, which could mainly be reflected in ischemic stroke patients but not in patients with hemorrhagic stroke. A lower serum vitamin D level was correlative with the poor prognosis of stroke.

## Graphical abstract

PRISMA flow chart of study selection. Articles included in the review met all inclusion criteria and with satisfactory methodological validity and clarity (n = 27).

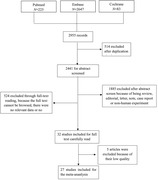

## 1 INTRODUCTION

Stroke is the second predominant cause of death and the key factor leading to disability in the world, and it affects a population of about 13.7 million people and causes approximately 5.5 million deaths each year (Campbell et al., 2019). Approximately 87% of strokes are caused by ischemic cerebral infarction, with the incidence of stroke being most prevalent in developing nations. Additionally, hemorrhagic stroke comprises approximately 10–15% of all strokes and has been reported to be related to a high mortality rate. From 1990 to 2016, the incidence rate of stroke increased in countries with low or middle‐income levels, while countries with high income levels experienced a decline of 42% in the same period (Roger et al., 2011). Despite a decrease in stroke prevalence, the socioeconomic burden of stroke rises due to factors, such as age, gender, and geographic location of affected individuals (GBD 2016 Stroke Collaborators, 2019). The burden of public health in China is also significant, as per a 2019 national population survey conducted within the Chinese population, and it is one of the most burdensome diseases in the world (Tu & Wang, 2023; Tu et al., 2023; Wang et al., 2022).

1,25‐dihydroxyvitamin D (25‐OHD) is an active form of vitamin D (VD) that includes two types of vitamins: D_2_ and D_3_. Vitamin D_2_, which comprises the steroid ergo sterol, is produced when plants, fungi, and yeast are exposed to UVB radiation. Vitamin D_3_ is formed through skin contact, during which the conversion of 7‐dehydrocholesterol into vitamin D_3_ occurs (Aspell et al., 2018; Tripkovic et al., 2012). Vitamin D_3_ (D_3_), namely, cholecalciferol, is mainly obtained through skin exposure to sunlight radiation. In general, sunlight exposure stimulates the skin to primarily synthesize vitamin D. However, the efficiency of sunlight action on the skin is influenced by various factors, including time of day, seasons, sunscreen usage, age, and other elements (Kennel et al., 2010). A comparison of different research results indicates that plasma 25‐OHD levels decrease with the higher latitudes in high‐latitude regions. However, skin pigmentation, temperature, and clothing can also affect UVB exposure (Andersen et al., 2020). Vitamin D is an elemental fat soluble vitamin that works a significant role in skeletal system. Besides the skeleton system, vitamin D also makes very significant effect on other organs and tissues (DeLuca, 2004). Furthermore, prior investigations have indicated a connection between insufficiency of vitamin D and the occurrence of rickets, osteomalacia, osteoporosis, dermatological disorders, as well as cardiovascular disease (CVDs) (Holick, 2007; Norman & Powell, 2014; Wadhwa et al., 2015).

Scientific research findings have indicated that an inadequate presence of vitamin D can increase the likelihood of experiencing a stroke (Judd et al., 2016; Talebi et al., 2020; Wang et al., 2021; Zhou et al., 2018). Furthermore, the outcomes for individuals affected by stroke and presenting with insufficient levels of serum vitamin D tend to be unfavorable (Hu et al., 2019; Kim et al., 2020; Wang et al., 2021). However, a few of studies have presented that vitamin D deficiency is not a cause of increased risk of stroke (Skaaby, 2015). In addition, on the basis of a report that a lack of vitamin D is related to an increased risk of stroke‐associated adverse events in China (Wei & Kuang, 2018). The occurrence of stroke depends on the complex interaction among multiple factors. To date, several predictable and unpredictable risk factors have been detected. However, it is still essential to identify more predictable risk factors or biomarkers for the prevention of stroke.

The main aim of the study was to probe the impact of low levels of vitamin D on stroke risk and prognosis of stroke.

## 2 METHODS

### 2.1 Literature search

The study was carried out in accordance with the guidelines provided by the Preferred Reporting Items for Systematic Reviews and Meta‐Analyses (PRISMA) statement (Page et al., 2021). A thorough exploration of the databases (PubMed, Cochrane Library, and Embase) had been implemented until July 29, 2022 Medical subject terms and free text terms bound for retrieval. The retrieval strategy is presented in Table 1.

**TABLE 1:** Detailed search strategy for each database.

Database	Search strategy	Retrieved records
Pubmed	(Stroke [Mesh]) OR (strokes)) OR (Cerebrovascular Accident)) OR (CVA)) OR (Cerebrovascular Apoplexy)) OR (Apoplexy, Cerebrovascular)) OR (Vascular Accident, Brain)) OR (Brain Vascular Accident)) OR (Cerebrovascular Stroke)) OR (Stroke, Cerebrovascular)) OR (Apoplexy)) OR (Cerebral Stroke)) OR (Stroke, Cerebral)) OR (Stroke, Acute)) OR (Acute Stroke)) OR (Cerebrovascular Accident, Acute)) OR (Acute Cerebrovascular Accident)) AND ((“Vitamin D”[Mesh]) OR (Cholecalciferol)) OR (Ergocalciferols)) OR (25‐Hydroxyvitamin D 2)) OR (Dihydrotachysterol))	225
Cochrane library	(Stroke [Mesh]) OR (strokes)) OR (Cerebrovascular Accident)) OR (CVA)) OR (Cerebrovascular Apoplexy)) OR (Apoplexy, Cerebrovascular)) OR (Vascular Accident, Brain)) OR (Brain Vascular Accident)) OR (Cerebrovascular Stroke)) OR (Stroke, Cerebrovascular)) OR (Apoplexy)) OR (Cerebral Stroke)) OR (Stroke, Cerebral)) OR (Stroke, Acute)) OR (Acute Stroke)) OR (Cerebrovascular Accident, Acute)) OR (Acute Cerebrovascular Accident)) AND ((“Vitamin D”[Mesh]) OR (Cholecalciferol)) OR (Ergocalciferols)) OR (Dihydrotachysterol))	83
Embase	(‘cerebrovascular accident’/exp OR ‘stroke’ OR ‘Cerebrovascular Apoplexy’ OR ‘Acute Cerebrovascular Accident’) AND (‘vitamin D’/exp OR ‘ergocalciferol’ OR ‘25‐Hydroxyvitamin D 2' OR ‘25‐hydroxyvitamin D’ OR ‘cholecalciferol’) AND [english]/lim AND [embase]/Lim	2647

### 2.2 Eligibility criteria

In this study, only as following literatures have been included: (1) studies that were published in English; (2) studies conducted on human subjects; (3) studies based on case‐control design, or randomized controlled trial (RCT), cohort design; (4) involvement of clear inclusion criteria for acute ischemic stroke or hemorrhagic stroke; (5) diagnosis of patients with stroke by CT or MRI before treatment; (6) the article included keywords related to stroke, vitamin D, and prognosis; (7) availability of complete data. After reviewing the supplementary materials, studies with incomplete data and duplicate researches were excluded.

### 2.3 Quality evaluation

Each study was evaluated independently by two readers to determine the risk of bias, and discrepancies were solved by consulting with a third reviewer. The researches about case‐control and cohort studies were evaluated according to the Newcastle–Ottawa Scale (NOS). The specific content and scoring criteria are shown (Supplementary Doc 1). By following the scoring system of this scale, a score ranging from 4 to 6 points signifies a significant risk of bias, while a score ranging from 7 to 9 points indicates a minimal risk of bias (Lo et al., 2014; Stang, 2014). Cochrane risk‐of‐bias tool was adopted to assess RCTs studies (Higgins & Green, 2011).

### 2.4 Data extraction

Two readers were responsible for article selection and extracted relevant data independently. Discrepancies during this process were solved through productive discussions or by seeking the guidance of a third reviewer. The study selection was strictly adhered to the predefined criteria for inclusion and exclusion. Regarding the data extraction, the following information was meticulously collected: the primary author's name, the study title, the nation the research was implemented, study type, the size of the sample, the calculated odds ratio (OR) or relative risk (RR) along with its corresponding 95% confidence interval (CI), the adjustment variables considered, and the different stroke subtypes and their corresponding vitamin D statuses.

Two reviewers independently completed study selection and data extraction. Study selection strictly followed the inclusion and exclusion criteria. Funnel plot was used to assess publication bias.

### 2.5 Statistical analysis

RR was used in the Prospective cohort study and OR was used in the retrospective study and 95% CIs on the basis of the Mantel–Haenszel random‐effects model. Heterogeneity was evaluated by using the *I* ^2^ statistic and *Q* test. The random‐effects model was used for all analyses in this study. Stata 16.0 software (Stata Corp, College Station, TX, USA) was used for data analysis.

## 3 RESULTS

**FIGURE 1:**
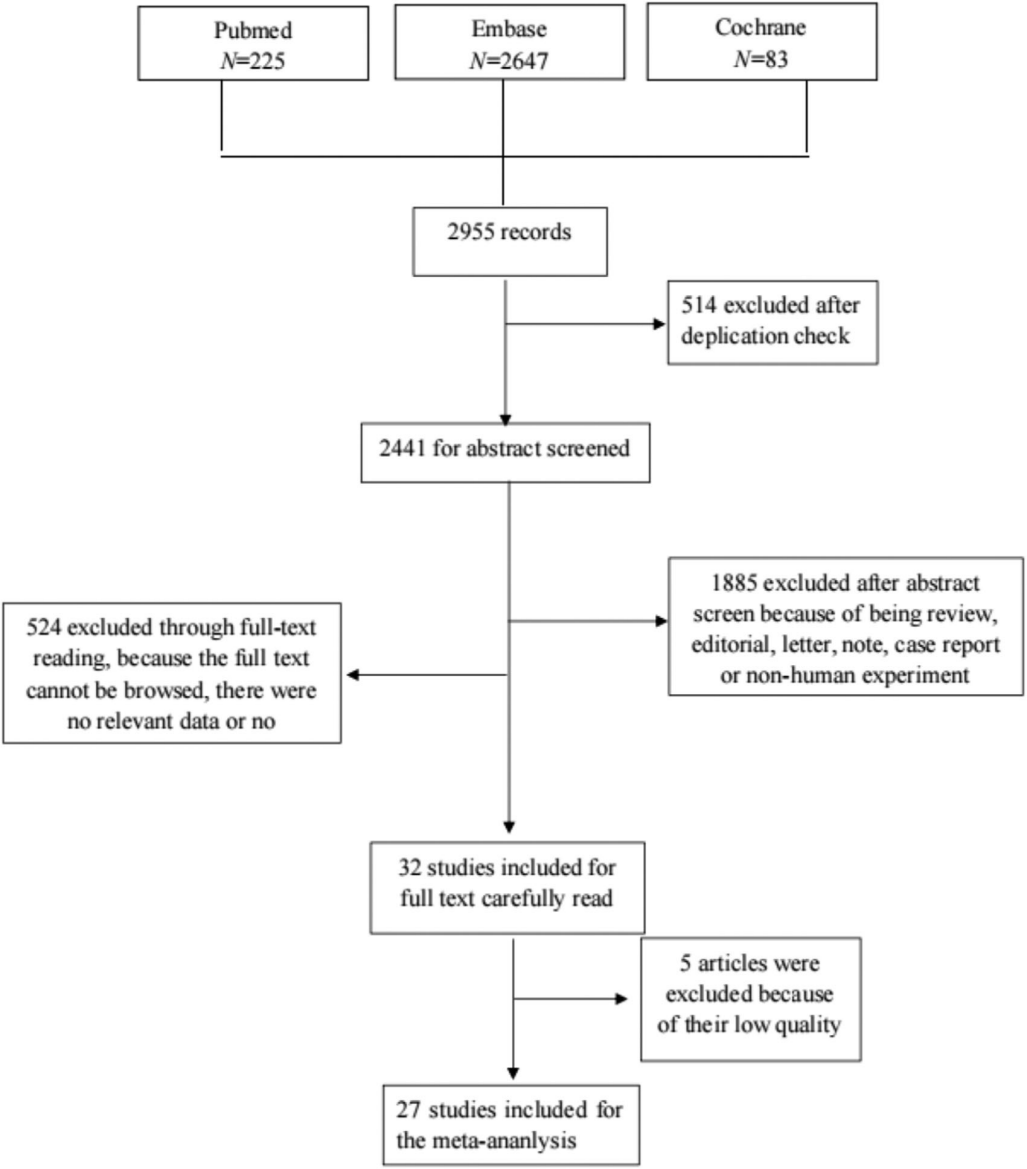
Flowchart of the study selection process.

Basic information of the included studies totally, 27 studies (Alfieri et al., 2017; Anderson et al., 2010; Bolland et al., 2009; Drechsler et al., 2010; Ford et al., 2014; Hu et al., 2019; Huang et al., 2019; Ji et al., 2017; Judd et al., 2016; Kim et al., 2020; Kojima et al., 2012; Kühn et al., 2013; Larsson et al., 2018; Leung et al., 2017; Marniemi et al., 2005; Michos et al., 2012; Perna et al., 2013; Schierbeck et al., 2012; Schneider et al., 2015; Skaaby et al., 2013; Sun et al., 2012; Szejko et al., 2022; Tan et al., 2017; Wei & Kuang, 2018; Xu et al., 2017; Xu et al., 2016; Zittermann et al., 2016) were finally involved in this study. The procedure of screening can be observed in Figure 1. A detailed search strategy for every database can be found in Table 1. The attributes of the incorporated trials and the demographic features of the patients are depicted in Table 2. Among all the included studies, 19 articles are cohort studies, 6 articles are case‐control studies, and 1 is RCT.

**TABLE 2:** Characteristics of the included studies.

Study	Country/district	Sample Size	Event	Number of cases	Sex male/female, no	Age, year	Major disease	Intake vitamin D	RR 95% CI
Drechsler et al. (2010)	Germany	1108	Stroke	89	598/510	66 ± 8	Diabetic hemodialysis	Not mentioned	2.58 (0.74‐8.98)
Marniemi et al. (2005)	Finland	755	Stroke	70	361/394	73.3	Not mentioned	Yes	1.13 (0.62–2.05)
Bolland et al. (2009)	New Zealand	1471	Stroke	59	0/1471	>55	No, postmenopausal women	No	1.4 (0.8–2.5)
Anderson et al. (2010)	USA	26,025	Stroke	208	6558/19,467	55 ± 21	Not mentioned	Not mentioned	1.78 (1.2–2.66)
Schierbeck et al. (2012)	Denmark	2013	Stroke	89	0/2013	50	No, postmenopausal women	Yes	1.68 (1.10–2.56)
Kojima et al. (2012)	Hawaii	7385	Stroke	960	7385/0	45–68	Not mentioned	Yes	1.22 (1.01–1.47)
			Ischemic stroke	651					1.27 (1.01–1.59)
			Hemorrhagic stroke	269					0.97 (0.68–1.38)
Sun et al. (2012)	USA	928	Ischemic stroke	464	0/928	30–55	No	Not mentioned	1.49 (1.01–2.18)
Perna et al. (2013)	Germany	7709	Stroke	354	3138/4571	50–74	No	Not mentioned	1.31 (0.95–1.81)
Kühn et al. (2013)	Germany	3115	Stroke	471	Not mentioned	35–65	Not mentioned	Not mentioned	1.25 (0.92–1.70)
Skaaby et al. (2013)	Denmark	8131	Stroke	316	4035/4096	55.4	Not mentioned	Not mentioned	0.88 (0.63–1.25)
Ford et al. (2014)	USA	5292	Stroke	309	811/4481	77.4 ± 5.6	Not mentioned	Yes	1.06 (0.8–1.32)
Zittermann et al. (2016)	Germany	154	Stroke	27	134/20	57–62	Left ventricular assist device implant	Not mentioned	2.44 (1.09–5.45)
			Ischemic stroke	13					2.36(0.78–7.19)
			Hemorrhagic stroke	14					1.91(0.67–5.46)
Tan et al. (2017)	China	404	Stroke	224	Not mentioned	62.46	No	No	12.92 (6.23–26.82)
			Ischemic stroke	121					11.67 (4.82–28.27)
			Hemorrhagic stroke	103					14.67 (5.38–40.02)
Judd et al. (2016)	USA	1547	Ischemic stroke	610	774/773, Half White, half Black	65–74	No	Not mentioned	1.84 (1.14–2.97)
			Hemorrhagic stroke	74					1.82 (0.91–3.65)
Larsson et al. (2018)	Sweden	43,8847	Ischemic stroke	34,217	Not mentioned	not mentioned	Not mentioned	Not mentioned	1.01 (0.94–1.08)
Michos et al. (2012)	USA	7981	Stroke	176	3654/4326, White: 5001, Black: 2980	≥30	Not mentioned	Not mentioned	1.74 (0.94–3.20)
				White person: 116					2.13 (1.01–4.50)
				Black person: 60					0.93 (0.49–1.80)
Schneider et al. (2015)	USA	12,158	Stroke	804	5228/6930, White: 9362, Black: 2793	57	Atherosclerosis	Not mentioned	1.34 (1.06–1.71)
Szejko et al. (2022)	USA	3026	Hemorrhagic stroke	1545	1362/1664	67	Not mentioned	Not mentioned	1.60 (1.05–2.43)
Wei and Kuang 2018,	China	266	Stroke	149	145/121	54–65	Not mentioned	Not mentioned	3.20 (1.70–4.20)
Huang et al. (2019)	China	82,464	Stroke	2982	32,172/50,292	51.4	Not mentioned	Not mentioned	1.04 (0.99–1.10)
			Ischemic stroke					0.99 (0.93–1.07)	
			Hemorrhagic stroke					1.09 (1.01–1.18)	
Leung et al. (2017)	China	3458	Ischemic stroke	244	1301/ 2157	63.2 ± 10.2	Osteoporosis	Not mentioned	1.74 (1.14–2.64)
Hu et al. (2019)	China	478	Ischemic stroke	136	250/228	62.8	No	Not mentioned	2.622 (1.226–5.641)
Kim et al. (2020)	Korea	328	Stroke	158	194/134	67.4 ± 13.2	Not mentioned	Not mentioned	3.38 (1.24–9.18)
Alfieri et al. (2017)	Brazil	286	Stroke	168	153/133	67.9 ± 1.0	Not mentioned	No	16.64 (5.66–46.92)
Ji et al. (2017)	China	277	Stroke	Recurrence: 31	166/111	65	No	No	2.55 (1.38–3.96)
				Prognosis: 84					3.03 (1.65–4.12)
Xu et al. (2017)	China	3041	Stroke	283	1975/1066	62.3	No	no	1.58 (1.04–2.41)
Xu et al. (2016)	China	3181	Stroke	742	2056/1125	62.3	No	No	2.24 (1.22–4.12)

### 3.1 Connection about vitamin D and risk of stroke

**FIGURE 2:**
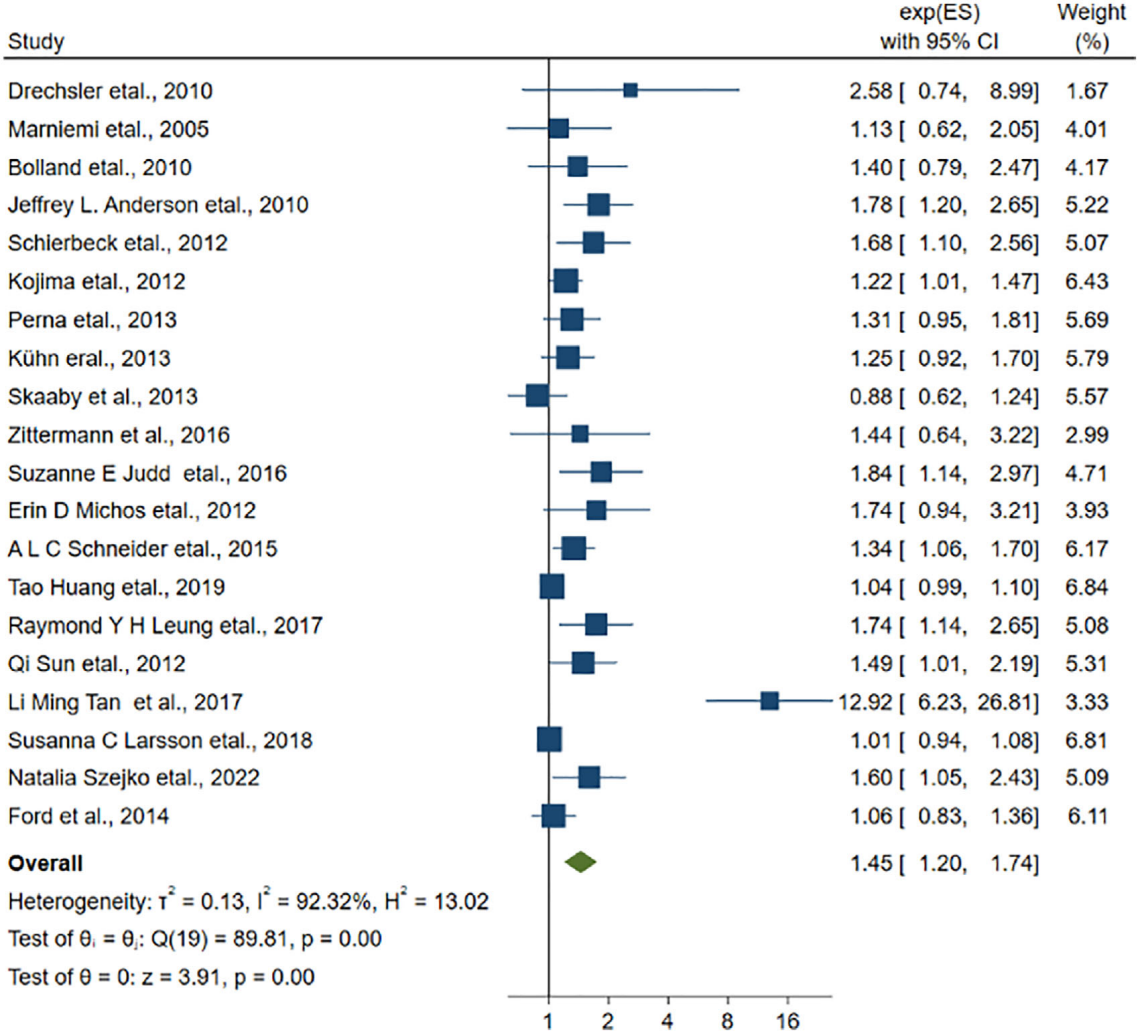
The linkage between vitamin D and the stroke.

Among all studies, 20 articles were related to the connection about the vitamin D level and the risk of stroke. The RR of stroke is calculated based on the comparison between individuals with the lowest and highest levels of vitamin D in their bloodstream. The combined RR was 1.45 (95% CI: 1.20–1.74, Figure 2). Besides, the *I* ^2^ was 92.32%, signifying the presence of considerable heterogeneity among the studies included in the analysis.

### 3.2 Connection about vitamin D and prognosis of stroke

**FIGURE 3:**
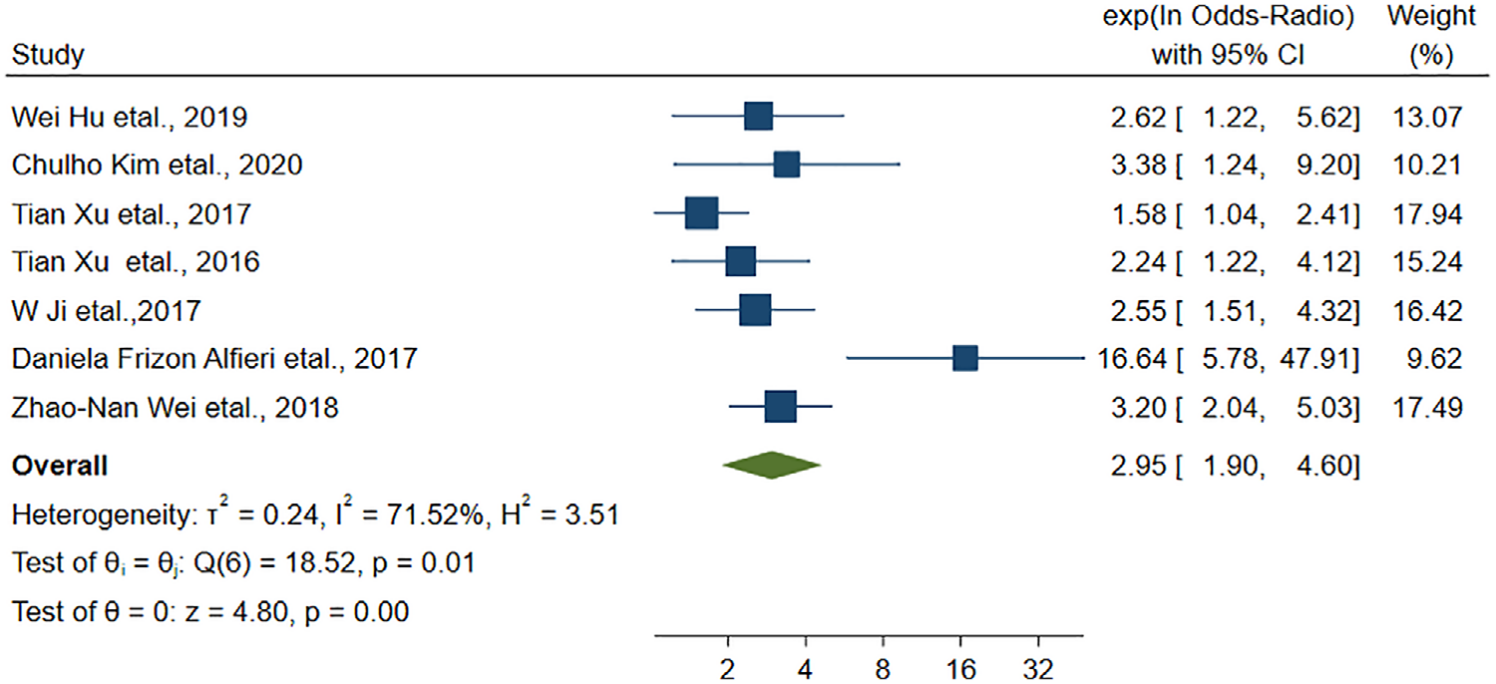
The linkage between vitamin D and the prognosis of stroke.

It is noteworthy to mention that seven articles have provided evidence suggesting a link between low levels of vitamin D in the blood and poor prognosis in individuals with ischemic stroke. The pooled RR for this association is found to be 2.95 (95% CI: 1.90–4.60, Figure 3).

### 3.3 Subgroup analysis

Of the 20 articles related to the connection about low vitamin D and the risk of stroke, there were 7 articles related to ischemic stroke, 6 articles related to hemorrhagic stroke, and 11 articles did not specify the type of stroke.

**FIGURE 4:**
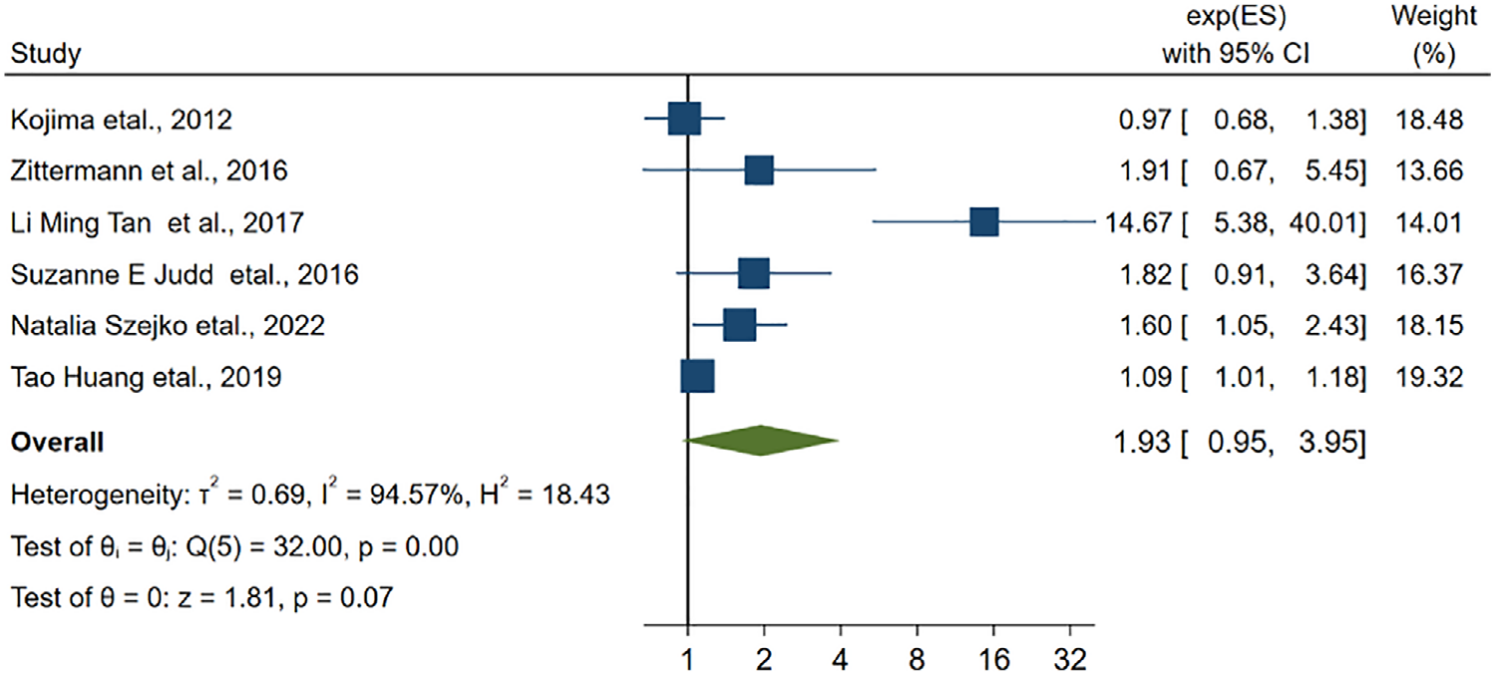
The linkage between vitamin D and the hemorrhagic stroke.

**FIGURE 5:**
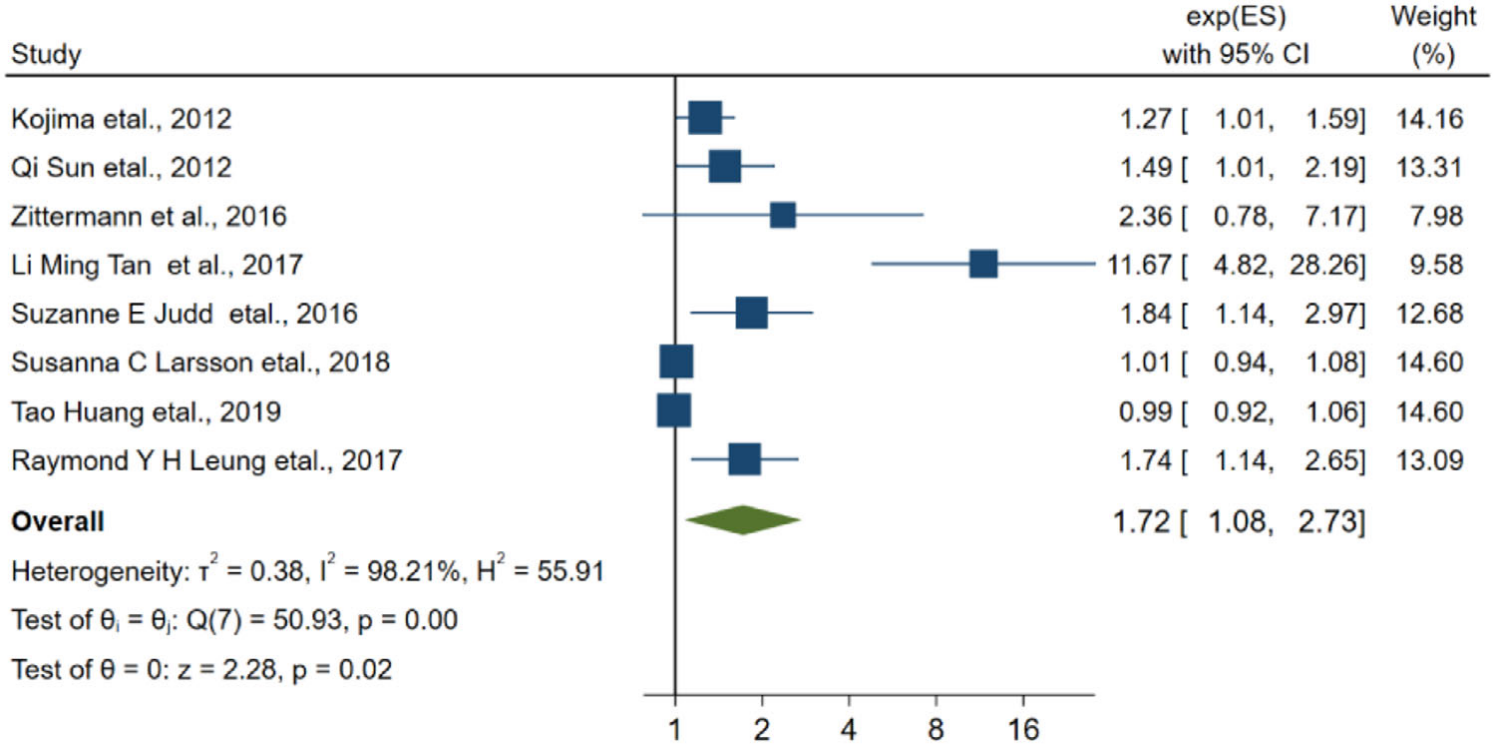
The linkage between vitamin D and the ischemic stroke.

**FIGURE 6:**
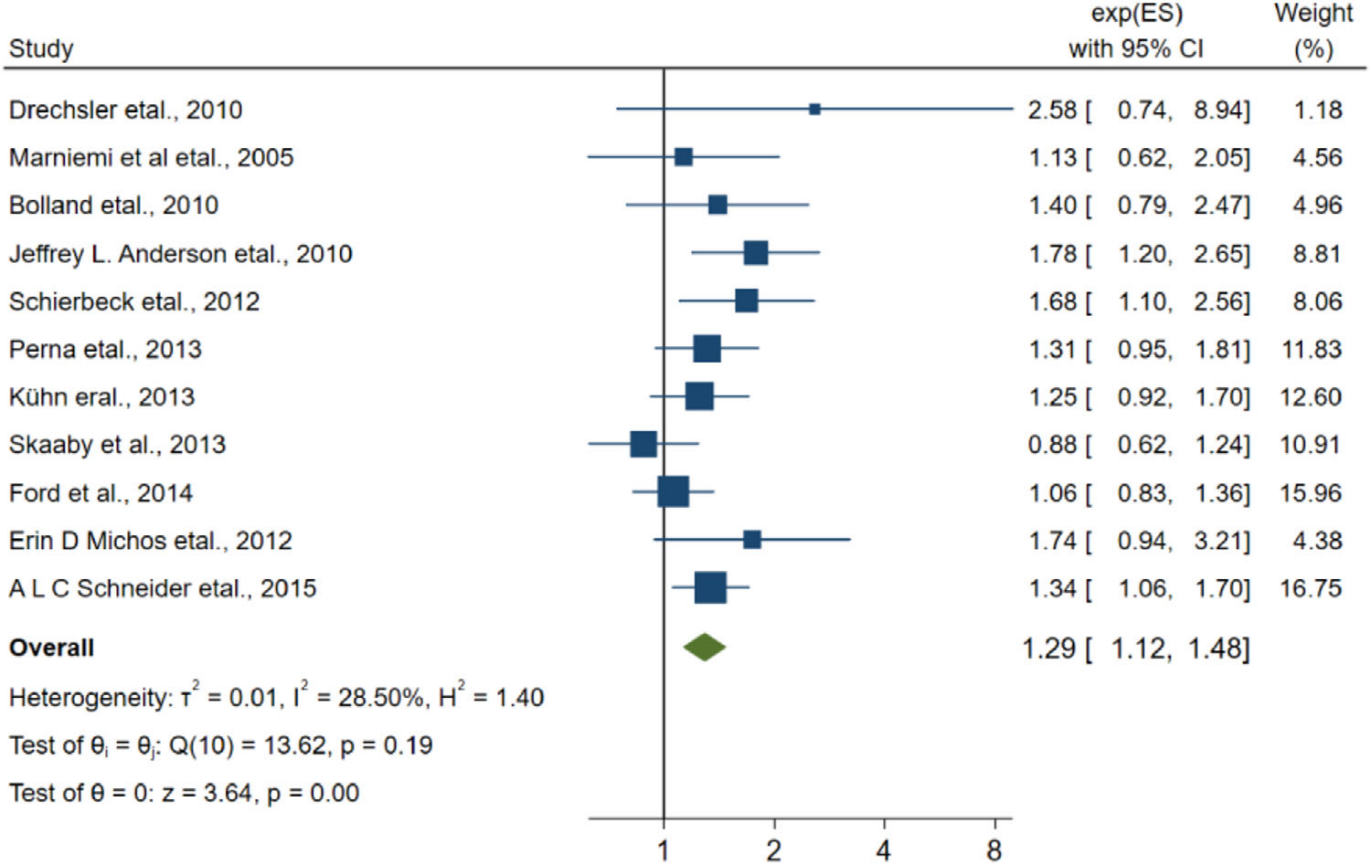
The linkage between vitamin D and the stroke without specific type.

Our research team presented 6 distinct articles regarding hemorrhagic stroke for separate and thorough analysis, and the combined RR was 1.93 (0.95–3.95, Figure 4). Besides, the *I* ^2^ was 94.57%, signifying the presence of considerable heterogeneity among the studies included in the analysis. We also analyzed 7 articles related to ischemic stroke, and the combined RR was 1.72 (1.08–2.73, Figure 5, *I* ^2 ^= 98.21). The results of the analysis of 11 other articles that did not determine specific type of stroke included the combined RR of 1.29 (1.12–1.48, Figure 6), and the *I* ^2^ was 28.50% < 50%.

**FIGURE 7:**
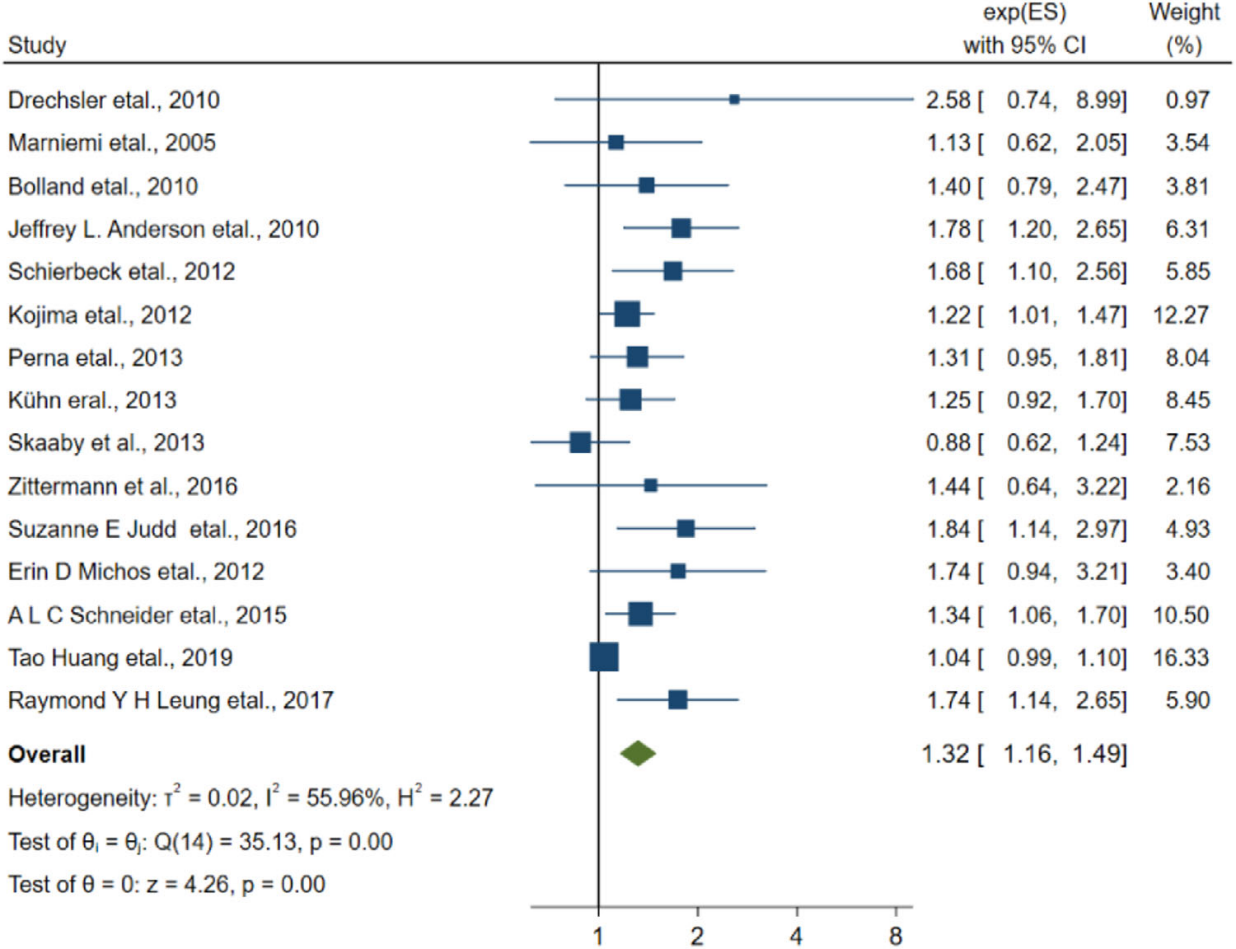
The linkage between vitamin D and the stroke for cohort study.

**FIGURE 8:**
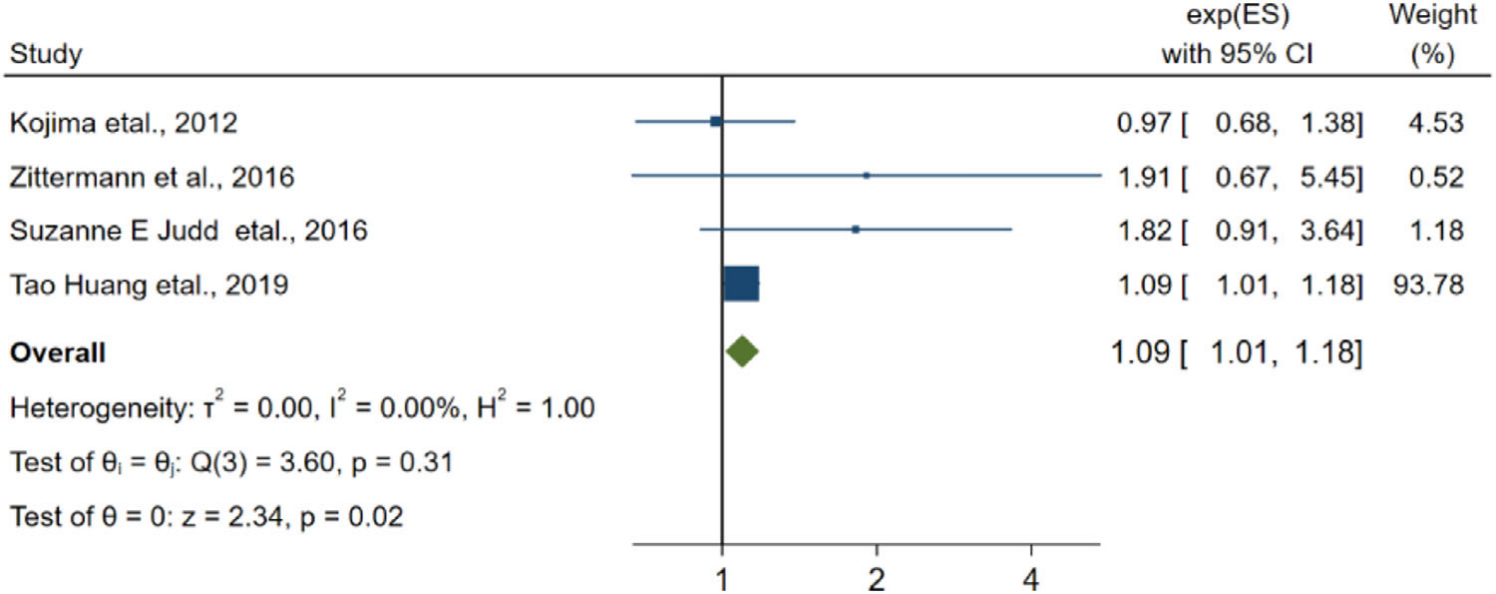
The linkage between vitamin D and the hemorrhagic stroke for cohort study.

**FIGURE 9:**
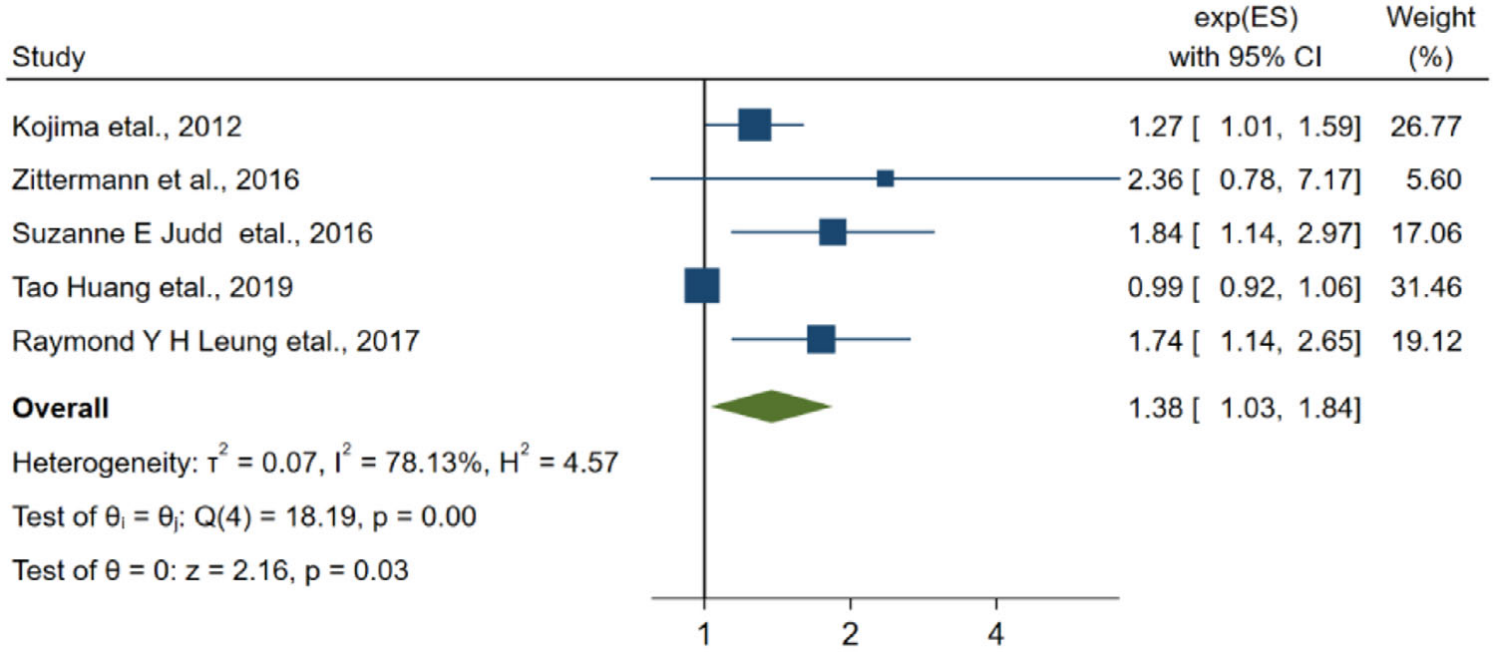
The linkage between vitamin D and the ischemic stroke for cohort study.

**FIGURE 10:**
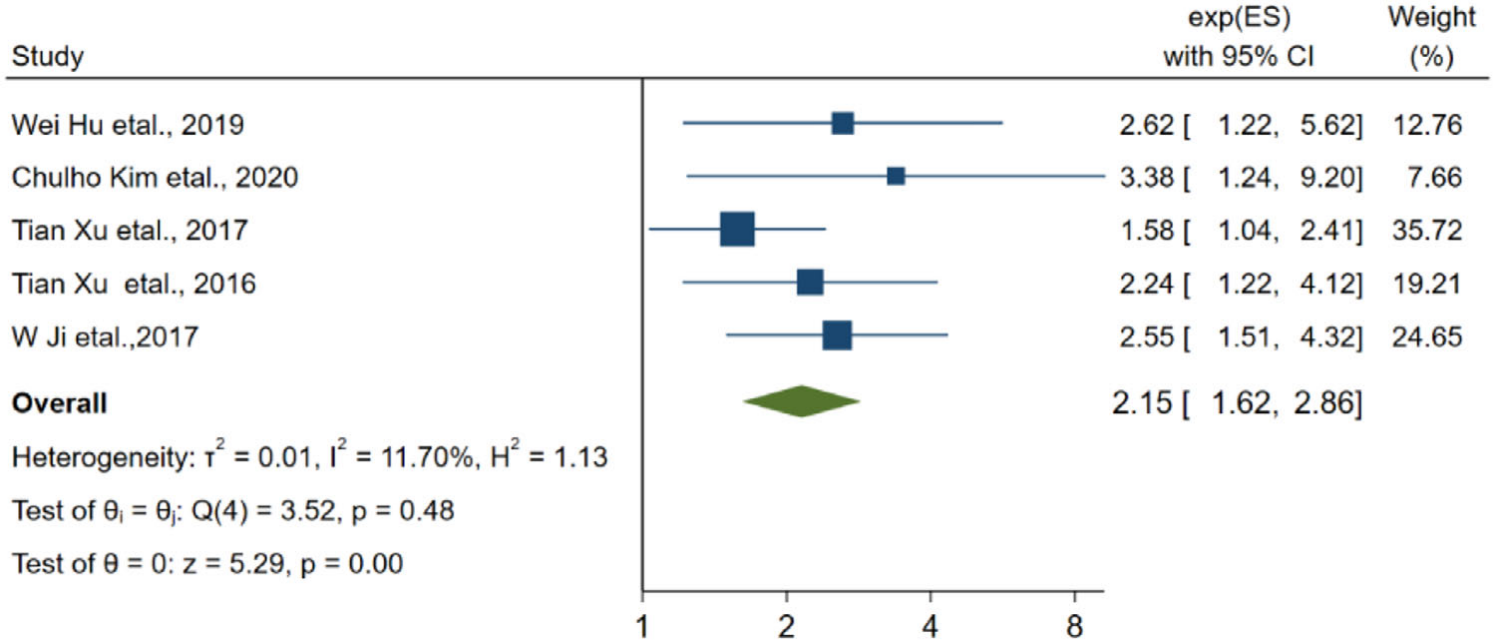
The linkage between vitamin D and the prognosis of stroke for cohort study.

In addition, due to their high heterogeneity, case‐control studies and RCTs were excluded based on the type of study. We implemented a separate analysis of cohort studies and conducted a source analysis of heterogeneity. It was found that if only cohort studies would be analyzed, their corresponding heterogeneity significantly decreased. The connections among vitamin D levels, the risk of stroke, the risk of hemorrhagic stroke risk, the risk of ischemic stroke, and prognosis of stroke are presented respectively in Figures 7, 8, 9, 10.

### 3.4 Assessment of risk of bias

The quality evaluation results of all researches are shown in Table 3.

**TABLE 3:** The results of the risk‐of‐bias assessment.

Study	Selection: a. Representativeness of the exposed cohort, 1 point; b. Selection of the nonexposed cohort, 1 point; c. Ascertainment of exposure, 1 point; d. Demonstration that outcome of interest was not present at start of study, 1 point	Comparability: a. Either exposed and nonexposed individuals must be matched in the design and/or confounders must be adjusted for in the analysis, 2 points	Outcome: a. Assessment of outcome, 1 point; b. Was follow‐up long enough for outcomes to occur, 1 point; c. Adequacy of follow‐up of cohorts, 1 point	Total score
Cohort study				
Drechsler et al. (2010)	3	2	3	8
Marniemi et al. (2005)	4	1	3	8
Bolland et al. (2009)	4	2	3	9
Anderson et al. (2010)	4	2	3	9
Schierbeck et al. (2012)	3	2	3	8
Kojima et al. (2012)	4	2	3	9
Perna et al. (2013)	4	2	3	9
Kühn et al. (2013)	4	2	3	9
Skaaby et al. (2013)	3	1	3	7
Zittermann et al. (2016)	3	1	2	6
Judd et al. (2016)	4	2	3	9
Michos et al. (2012)	4	2	3	9
Schneider et al. (2015)	4	2	3	9
Huang et al. (2019)	4	2	3	9
Leung et al. (2017)	4	2	3	9
Hu et al. (2019)	4	2	3	9
Kim et al. (2020)	4	1	3	8
Xu et al. (2017)	4	1	3	8
Xu et al. (2016)	4	1	3	8
Ji et al. (2017)	4	1	3	8

Study	Selection: a. Is the case definition adequate, 1 point; b. Representativeness of the cases, 1 point; c. Selection of controls, 1 point; d. Definition of controls, 1 point	Comparability: a. Comparability of Cases and controls on the basis of the design or analysis, 2 point	Exposure: a. Ascertainment of exposure, 1 point; b. Use the same method to determine exposure factors for both the case and control groups, 1 point; c. Nonresponse rate, 1 point	Total score
Case‐control study				
Sun et al. (2012)	4	2	2	9
Tan et al. (2017)	4	2	3	9
Larsson et al. (2018)	4	1	3	8
Szejko et al. (2022)	4	2	3	9
Alfieri et al. (2017)	4	2	3	9
Wei and Kuang (2018)	4	1	3	8

Study	Random allocation method	Allocation concealment	Blinding	Integrity of results	Selective research report results	Other sources of bias	Total score
RCT							
Ford et al. (2014)	1	1	1	1	1	1	6

### 3.5 Sensitivity analysis

As a result of a high heterogeneity, RCT and case‐control studies were excluded according to the type of study, for the sample size of case‐control studies and RCTs was small. We implemented a separate analysis of the cohort study. Regarding the linkage about vitamin D and the risk of stroke, the RR of the studies was 1.45 (95% CI: 1.14–1.41), and *I* ^2^ was 92.32% > 50%, in which after excluding five studies (Ford et al., 2014; Sun et al., 2012; Tan et al., 2017; Xu et al., 2017; Xu et al., 2016), the merged RR of the remaining studies was 1.32 (95% CI: 1.16–1.49), and *I* ^2^ was 55.96% > 50%, using a random‐effects model. According to the linkage about vitamin D level and the risk of ischemic stroke, the RR of the studies was 1.72 (95% CI: 1.08–2.73), and *I* ^2^ was 98.21% > 50%, in which after excluding three studies (Larsson et al., 2018; Sun et al., 2012; Tan et al., 2017), the RR of the residual researches was 1.38 (95% CI: 1.03–1.84), *I* ^2^ was 78.13% > 50%. Subsequently, studies with a high proportion of female participants were excluded, and the RR changed. After excluding three studies (Bolland et al., 2009; Huang et al., 2019; Schierbeck et al., 2012), the RR indicated that there was a 1.34‐fold (95% CI: 1.18–1.51) increased risk of stroke related to low serum vitamin D levels, *I* ^2^ was 21.69% < 50%. Similarly, after excluding one study (Huang et al., 2019), the RR between low serum vitamin D level and the risk of ischemic stroke was 1.54 (95% CI: 1.19‐1.99), and *I* ^2^ was 31.73% < 50%. Of the 27 articles, 2 of them (Drechsler et al., 2010; Leung et al., 2017) involved participants with osteoporosis and diabetic hemodialysis. And four of them (Ford et al., 2014; Kojima et al., 2012; Marniemi et al., 2005; Schierbeck et al., 2012), participants had a history of taking vitamin D. After excluding studies on major illnesses and vitamin D intake, the RR of increased risk of ischemic stroke caused by low levels of vitamin D was 1.86, and *I* ^2^ was 99.41% > 50%, the RR of increased risk of stroke without specific type caused by low levels of vitamin D was 1.30, and *I* ^2^ was 28.71% < 50%, the RR between reduced vitamin D level and the risk of hemorrhagic stroke was 2.28, and *I* ^2^ was 92.25% > 50%.

Regarding the linkage about low vitamin D level and prognosis of stroke, after excluding two study (Alfieri et al., 2017; Wei & Kuang, 2018), the RR of the residual researches was 2.15 (95% CI: 1.62–2.86 3.15), *I* was 11.70% < 50%, and a fixed‐effect model was utilized.

### 3.6 Assessment of publication bias

**FIGURE 11:**
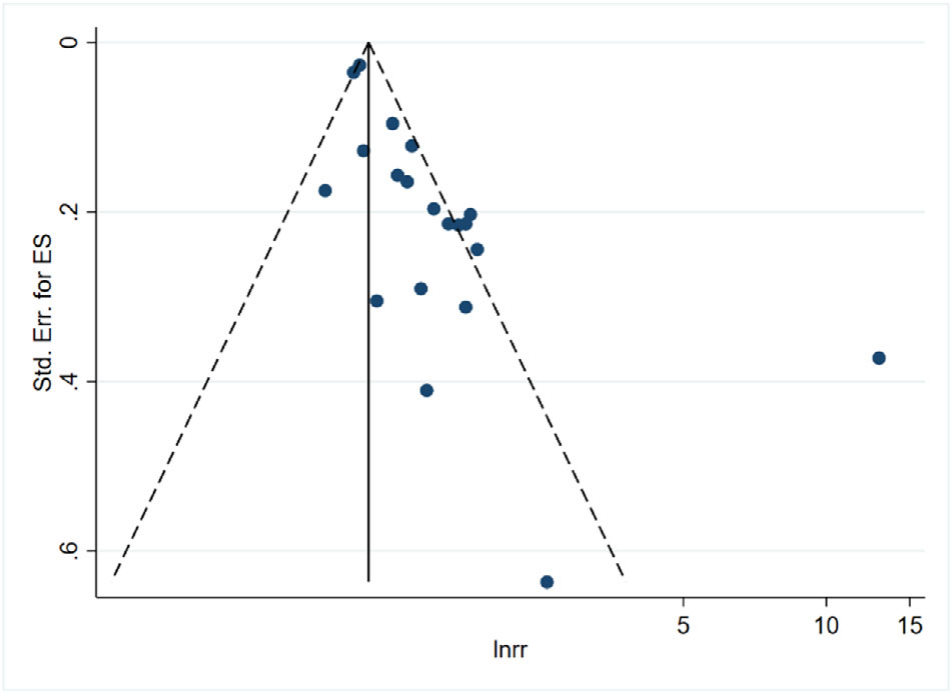
Linkage between vitamin D and the risk of stroke. It is noteworthy that the distribution of points for each study appears to be asymmetric, which could suggest the possibility of publication bias.

**FIGURE 12:**
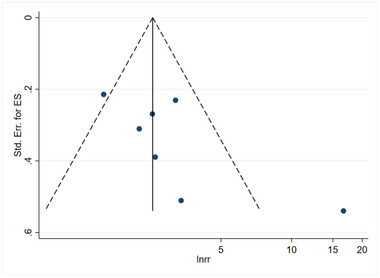
Linkage between vitamin D and prognosis of stroke. It is noteworthy that the distribution of points for each study appears to be asymmetric, which could suggest the possibility of publication bias.

**FIGURE 13:**
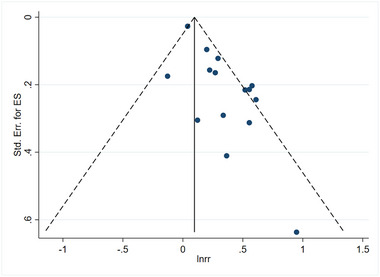
Linkage between vitamin D and the risk of stroke for cohort study. After excluding case‐control and randomized controlled studies, the remaining studies were distributed symmetrically on the funnel plot.

**FIGURE 14:**
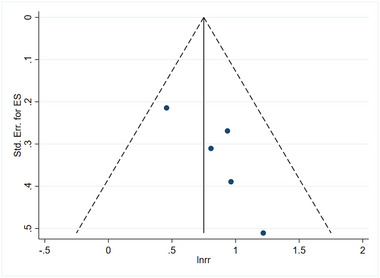
Linkage between serum vitamin D levels and prognosis of stroke for cohort study. After excluding case‐control and randomized controlled studies, the remaining studies were distributed symmetrically on the funnel plot.

Figures 11 and 12 illustrate the publication bias concerning the linkage about serum vitamin D levels and the risk of stroke, as well as the prognosis associated with stroke. Furthermore, funnel plots were generated using Stata 16.0 software to assess publication bias. Dissymmetry in the funnel plot indicated potential publication bias. When searching for the source of bias, it is worth noting that after excluding case‐control studies and RCTs, the remaining studies were distributed symmetrically on the funnel plot. It was found that the number of participants of case‐control studies and RCTs was small, which may be the reason for publication bias. If only the cohort study is analyzed, the publication bias of the linkage about vitamin D level and the risk of stroke has significantly changed, as shown in Figure 13. Similarly, if only the cohort study is analyzed, the publication bias of the linkage about vitamin D level and prognosis of stroke has also changed significantly, as shown in Figure 14.

## 4 DISCUSSION

Stroke is a prevalent acute cerebrovascular disease, serving as the leading contributor to impairment among adults in China. At present, stroke prevention is regarded as the most effective measure. In addition to the controllable and uncontrollable risk factors currently known, it is essential to further explore controllable risk factors that are to prevent stroke.

The study contained 27 studies that probed the linkage about vitamin D level and the stroke onset. Among them, 20 articles focus on whether low concentration vitamin D raise stroke risk. Through the combined risk of 20 studies, it was found that low concentrations vitamin D might raise the risk of stroke. Besides, seven studies have been associated with the linkage about serum vitamin D and prognosis of stroke. By calculating comprehensive risk, a positive correlation was identified between low concentrations vitamin D and poor prognosis of stroke. According to the analysis of 20 studies, it was found that there was a certain degree of connection among vitamin D level, the risk of ischemic stroke, the risk of hemorrhagic stroke, and the risk of unspecified stroke. Therefore, subgroup analysis was conducted; it was revealed that there was no apparent correlation between low vitamin D level and the risk of hemorrhagic stroke, while there was a correlation among low vitamin D level, the risk of ischemic stroke, and the risk of unspecified stroke.

However, a high degree of heterogeneity among the studies included in this study was inevitably. To make the conclusions more convincing and scientific, the sources of heterogeneity were explored. Case‐control studies and RCTs were excluded. Studies of the connection between concentrations vitamin D and prognosis of stroke, and studies on the connection between concentrations vitamin D and the risk of hemorrhagic stroke, the heterogeneity was significantly reduced and *I* ^2^ was < 50% after excluding case‐control studies and RCTs. Similarly, in studies on the correlation between serum vitamin D level and the risk of stroke and the relationship between concentrations vitamin D and the risk of ischemic stroke, the heterogeneity was significantly reduced after excluding case‐control studies and RCTs, although *I* ^2^ was > 50%. Through subgroup analysis, it was found that the number of participants of case‐control studies and RCTs was low. Through sensitivity analysis, it was revealed that after excluding case‐control studies, RCTs, and a high proportion of women, the heterogeneity of each subgroup significantly decreased. Therefore, sample size and gender are the main sources of heterogeneity. After excluding studies on major illnesses and vitamin D intake, there was evident that no significant changes were found in heterogeneity in this study. In addition, individuals’ dietary habits in different regions are also different, leading to differences in vitamin D intake. Furthermore, participants’ age also be the reason for the heterogeneity. Numerous research investigations have indicated that the intake of VD can enhance the adverse consequences associated with stroke (Kadri et al., 2020; Narasimhan & Balasubramanian, 2017).

The human body has the ability to synthesize vitamin D through the exposure of ultraviolet rays on two of its precursors, namely 7‐dehydrocholesterol and ergosterol (Catharine Ross et al., 2011). To our knowledge, vitamin D causes kinds of impacts on some systems, mainly including endocrine system, skeleton and cardiovascular system (Tripkovic et al., 2012). VD plays as a helpful role on the nervous system (Huang et al., 2016; Kiraly et al., 2006; Turetsky et al., 2015), including enhancing synaptic plasticity (Pertile et al., 2016), alleviating oxidative stress (Won et al., 2015), thus reducing brain injury and inflammatory reactions in the brain (Evans et al., 2018; Nissou et al., 2014). In addition, maintaining blood–brain barrier integrity and reducing damage are key ingredients of effective intervention measures for ischemic stroke. Therefore, the injury of blood–brain barrier will aggravate the brain injury (Won et al., 2015). Previous studies indicated that cerebral ischemia‐reperfusion injury can lead to the destruction of the structure and function of the BBB, resulting in an increase in BBB permeability (Latour et al., 2004), and the more severe and longer the duration of the BBB injured, the more severe the brain injury (Chen et al., 2009). Research showed that therapy with vitamin D can prevent BBB injury. Some researches indicated that VD can to some extent inhibit ischemic damage to brain endothelial cells. Due to the important role of endothelial cells in protecting the brain, vitamin D has a certain protective effect on endothelial function (Won et al., 2015). In the present research, intake of calcitriol for 7 days dramatically reduced the infarct scope and was associated with the raised activities of NR3A and phosphorylated cAMP‐responsive element binding protein (p‐CREB), following cerebral ischemia/reperfusion injury. The protective role on the nerves was weakened by cotreatment with PD98059, an MEK (the upstream kinase of ERK) inhibitor (Fu et al., 2013). Another study showed that VD safeguard rats from middle cerebral artery occlusion/reperfusion injury by preventing BBB disruption through activation of the anti‐inflammatory peroxisome proliferator‐activated receptor‐gamma(PPAR‐γ) pathway and upregulation of brain‐derived neurotrophic factor (Guo et al., 2018). This may be explaining why low concentration vitamin D level raise the risk of ischemic stroke and the risk of poor prognosis of stroke. No consistent phenomenon was found in a clinical trial, and vitamin D supplementation did not lead to a importantly amelioration in outcomes after stroke (Rist et al., 2021). Experiments conducted on animals have demonstrated that the presence of a diminished level of vitamin D did not pose as a risk element towards the negative prognosis associated with stroke (Evans et al., 2018). Kaul and Manikinda (2021) demonstrated that VD level could work in the risk of stroke, while it could not be regarded as a risk factor for stroke. Fu et al.’s (2022) research indicated that no reduction in stroke risk was observed through supplementary vitamin D intake. Many factors can lead to differences in the effectiveness of supplementing the same dose of vitamin D, such as gender, underlying diseases, and duration of exposure to sunlight (Kennel et al., 2010). Due to the different conclusions reached in these four studies, we reviewed the literature and found that the number of the four researches participants was relatively small.

The present meta‐analysis mainly analyzed the linkage among vitamin D, the risk of stroke and prognosis of stroke, which was not assessed in previous studies. At the present research, there was a phenomenon that VD could play different roles in stroke subtypes. Although several studies have consistently demonstrated that a deficiency in vitamin D at low levels might increase the likelihood of experiencing a hemorrhagic stroke, vitamin D supplementation could improve the prognosis of hemorrhagic stroke (Ashouri et al., 2021). In line with preceding research, the investigation revealed that insufficient vitamin D could potentially elevate the likelihood of encountering ischemic stroke (Zhou et al., 2018). In contrast to prior investigations, this particular study additionally suggests that inadequate vitamin D levels could potentially result in an adverse prognosis among individuals suffering from stroke. Taken together, in our present investigation, we revised previous meta‐analyses by incorporating recently published research. Additionally, in order to examine the variation among these studies, we performed additional subgroup analyses. Further investigation is required to validate the linkage between vitamin D and the likelihood of hemorrhagic stroke. However, our meta‐analysis has some shortcomings. First, there was a high degree of heterogeneity in this study. Second, the funnel plot revealed that there was an obvious publication bias.

## 5 CONCLUSION

In conclusion, lower concentration vitamin D was found to be related to an increased risk of stroke, which could mainly be reflected in ischemic stroke patients but not in patients with hemorrhagic stroke. A lower serum vitamin D level was correlative with the poor prognosis of stroke. Further investigation is required to validate whether reduced serum vitamin D levels contribute to an elevated susceptibility to hemorrhagic stroke, and if vitamin D supplementation can mitigate the risk of stroke and enhance the prognosis associated with this condition. In‐depth analysis and exploration of the impact of dietary habits on vitamin D intake is needed in future study.

## AUTHOR CONTRIBUTIONS

**Jianrong Xiong**: Conceptualization; investigation; validation. **Chenliang Zhao**: Methodology; software; data curation. **Jinhui Li**: Validation; formal analysis; supervision; funding acquisition; writing—review and editing; writing—original draft. **Yongxiang Li**: Resources; Writing—review and editing.

## FUNDING

JRX received funding from the Major Science and Technology Research Program of Jinhua City (2021‐4‐271) and CLZ received funding from the General Research Projects of Zhejiang Provincial Department of Education (Y202044752). The funders had no role in the study design, data collection and analysis, decision to publish, or preparation of the manuscript.

## CONFLICT OF INTEREST STATEMENT

The authors declare that they have no competing interests.

### PEER REVIEW

The peer review history for this article is available at https://publons.com/publon/10.1002/brb3.3577.

## Supporting information

Supporting Information

## Data Availability

The datasets used and/or analyzed during the present study are available from the corresponding author upon reasonable request.
