# Supplementary material for: A systematic review and meta‐analysis of the linkage between low vitamin D and the risk as well as the prognosis of stroke
Source: Brain Behav. 2024 Jun 14;14(6):e3577. doi: 10.1002/brb3.3577 (PMC11177038; doi:10.1002/brb3.3577)
Supplement: Supplementary file 1 — Supporting Information [file BRB3-14-e3577-s001.docx]

Supplemental File 1: Newcastle Ottawa grading Scale (NOS)

**NEWCASTLE - OTTAWA QUALITY ASSESSMENT SCALE**

**Selection:** (Maximum 5 stars)

1) Representativeness of the sample:

1. Truly representative of the average in the target population. * (all subjects or random sampling)
2. Somewhat representative of the average in the target population. * (nonrandom sampling)
3. Selected group of users.
4. No description of the sampling strategy. 2) Sample size:
5. Justified and satisfactory. *
6. Not justified.
7. Non-respondents:
   1. Comparability between respondents and non-respondents characteristics is established, and the response rate is satisfactory. *
   2. The response rate is unsatisfactory, or the comparability between respondents and non-respondents is unsatisfactory.
   3. No description of the response rate or the characteristics of the responders and the non-responders.
8. Ascertainment of the exposure (risk factor):
   1. Validated measurement tool. **
   2. Non-validated measurement tool, but the tool is available or described.*
   3. No description of the measurement tool.

**Comparability:** (Maximum 2 stars)

1) The subjects in different outcome groups are comparable, based on the study design or analysis. Confounding factors are controlled.

1. The study controls for the most important factor (select one). *
2. The study control for any additional factor. *

**Outcome:** (Maximum 3 stars)

1. Assessment of the outcome:
   1. Independent blind assessment. **
   2. Record linkage. **
   3. Self report. *
   4. No description.
2. Statistical test:
   1. The statistical test used to analyze the data is clearly described and appropriate, and the measurement of the association is presented, including confidence intervals and the probability level (p value). *
   2. The statistical test is not appropriate, not described or incomplete.
